# Point cloud registration of arrester based on scale-invariant points feature histogram

**DOI:** 10.1038/s41598-022-21657-8

**Published:** 2022-10-31

**Authors:** Wen Zhu, Lingchao Chen, Beiping Hou, Weihan Li, Tianliang Chen, Shixiong Liang

**Affiliations:** School of Automation and Electrical Engineering, Zhejiang University of Science and Technology, Hangzhou, 310023 China

**Keywords:** Engineering, Electrical and electronic engineering

## Abstract

Arrester is an important lightning protection device in the electrical field. The parameters of arrester such as creepage distance, umbrella distance and diameter are important for product quality, but they are difficult to measure because the shape of arrester is irregular. However, the three-dimensional (3D) reconstruction technique is efficient in measuring arrester parameters. The uniform distributed structure of umbrella skirt on the arrester surface restrict the registration of point cloud. In this paper, a scale-invariant points features histogram (SIPFH) descriptor is proposed; the descriptor combines the characteristics of Scale-invariant Feature Transform (SIFT) and fast point feature histogram (FPFH). Moreover, the improved Levenberg-Marquardt (LM) algorithm is presented, the maximum distance of corresponding points in the iterative process is adjusted to realize the local optimization. The point cloud registration method consists of two parts: primary registration method based on SIPFH, and secondary registration method based on improved LM algorithm. Point clouds of different arresters are collected to establish datasets, some of which have interference. Experimental results indicate that the root mean square error of the method is less than 0.02 m; the average running time is 2.7 s, which is $41.4\%$ of the conventional method based on FPFH.

## Introduction

The lightning arrester is a kind of lightning protection device, whose function is protecting power facilities from lightning strikes. Arrester is widely used in overhead distribution lines^1,2^. However, the arrester will conduct electricity under non-lightning conditions when the creepage distance (the shortest distance along the surface of a solid insulating material between two conductive parts), umbrella distance (the shortest distance between the edges of two skirt structures) and other parameters are not standard. In severe cases, arrester may burst during operation. At present, the main measurement methods of arrester parameters depend on manual work^3,4^. While there are many problems such as inefficiency in time, heavier workload, and lower accuracy. Laser detection is another effective way for measurements, but it is difficult to realize due to the shielding in umbrella skirt area. 3D reconstruction technology is an effective tool to detect the surface structure of arresters. When the whole 3D information of the arrester is obtained, it is easy to calculate the creepage distance, umbrella distance, and other parameters.

The reconstruction results determine the measurement accuracy, and the point cloud registration is the key step in 3D reconstruction. The point cloud registration is the process of finding a spatial transformation that aligns two point clouds from different acquisition angles.

In recent years, point cloud registration has become an important research direction in the field of 3D measurement. Scholars have proposed a series of methods for point cloud registration in different applications. Iterative closest point (ICP) is the most popular example to achieve the registration^5^. The ICP aligns two sets of points iteratively until stopping conditions are satisfied through finding correspondences at point-wise level. When point cloud contains a large number of points, it may incur from excessive memory cost. The ICP is easy to fall into locally optimal solution. In order to solve the problems, Zhou^6^ proposed a fast global registration algorithm, which only optimizes for motion and uses the scans to evaluate the registration error. Kwok^7^ proposed a double standard space sampling technique, It realizes registration by measuring all points on the curve, which slow down the convergence of the estimated value and even get the wrong solution. To registrate the large-scale point clouds, Hong^8^ proposed a 3D-Normal Distribution Transformation (3D-NDT) algorithm, in which the referenced point clouds are represented by a set of Gaussian distributions with different probability density functions in NDT algorithm. Through the transformation between estimated reference point cloud and target point cloud, the registration qualities are evaluated and compared.

Rabbani^9^ proposed a registration method based on Levenberg-Marquardt (LM), which shows successful results to some extent by geometric constraints. Rusu^10^ proposed a method based on fast point feature histogram (FPFH). However, it takes too much memory to calculate FPFH descriptor for each point. Wang^11^ proposed Deep Closest Point (DCP), which learns to perform registration. Registration methods have thus far focused on point clouds and show some limitations. Zhong^12^ designed a corresponding extraction scheme based on a novel descriptor with texture encoding and geometric information, which cannot be applied to targets with unclear texture. In summary, conventional registration algorithms cannot meet our demands.

In this paper, the work is shown as follows:

1. A scale-invariant points feature histogram (SIPFH) descriptor is presented to describe structural characteristics of arrester;
2. An improved LM algorithm is proposed for local optimization of point cloud registration;
3. An effective method structure is established to estimate a rigid transformation aligning two point clouds.
4. 3D structural information of various arresters is captured by laser cameras to support the study;
5. The performance of the method has been evaluated by applying it to 3D data of different arresters.

## Materials and methods

The datasets are collected by laser camera of Azure Kinect DK. The camera parameters include narrow field of view depth mode, pixel $640 \times 576$ and system error $<11 \; \text{mm} + 0.1\%$ distance.

### Data collection

**Figure 1:**
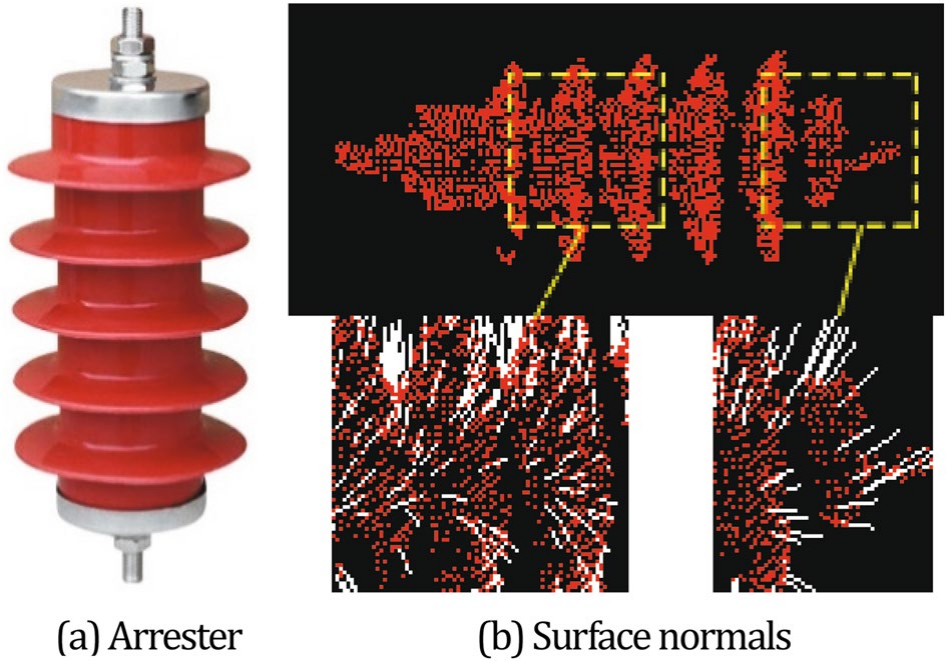
Typical arresters. From left to right: three different arrester models.

**Figure 2:**
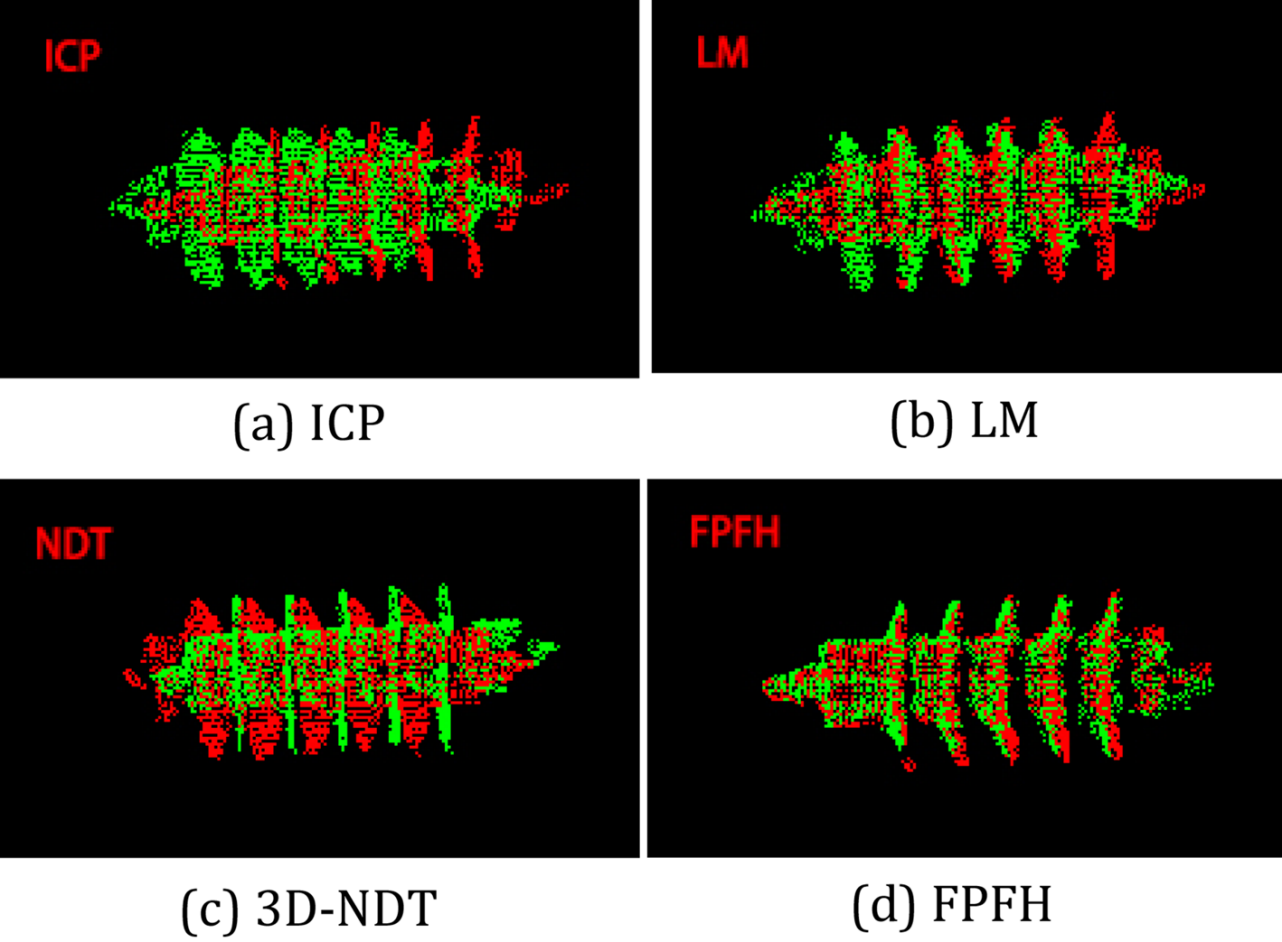
Arrester point cloud datasets. From left to right: there are 16 groups point clouds from three arrester point cloud datasets.

The experimental data come from three different zinc oxide arresters shown in Fig. 1. We collect 3D information of three arresters by laser camera and establish three datasets. Each dataset contains 200 groups of two point clouds, and some point clouds are mixed with interference objects. In addition, the transformation between rotation and translation of laser camera is measured in datasets. The 16 groups point clouds of each dataset are shown in Fig. 2.

Dataset 1: The data obtained from HY5WS-17/50 arrester (Fig. 1 left 1). The length is medium, the structure density of the umbrella skirt is low.

Dataset 2: The data obtained from HY5WS-10/30 type arrester (Fig. 1 left 2). The structure density of umbrella skirt is small, the length is minimum, and the arrester is accessible to misregistration.

Dataset 3: The data obtained from HY5WZ-17/45 type arrester (Fig. 1 left 3). The structure density of umbrella skirt is high, and the data is accessible to misregistration.

### Sample analysis of arrester

In this section, we study the structural characteristics of lightning arrester and discuss some conventional point cloud registration methods as preliminary study.

#### Arrester structure characteristics

**Figure 3:**
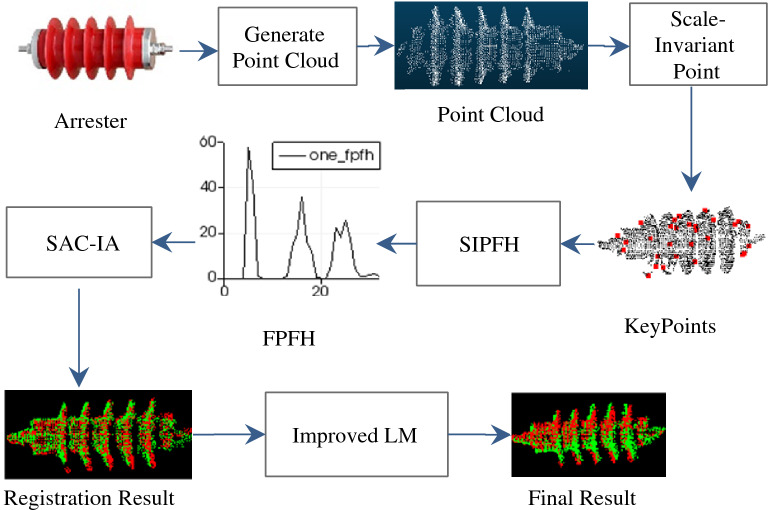
Structure characteristics of ZnO arrester. Figure (**a**) shows a typical arrester; The top figure of figure (**b**) shows the 3D point cloud information collected by laser camera from arrester in figure (**a**); The bottom figure of figure (**b**) shows the normals of some point clouds in the yellow box from the top figure.

The typical arrester is shown in Fig. 3a. In Fig. 3b, the top figure is the arrester point cloud collected by laser camera from the arrester in figure (a). In Fig. 3b, the bottom figure show surface normals of the yellow box in the arrester region. the surface normals are approximately estimated from the arrester point cloud data. It is an important attribute of geometry surface, which can directly represent the structural changes. The study support details of our arrester registration method.

This structure characteristics of the arrester include the following ones:

1. The appearance of arrester is symmetrical;
2. There’s a uniform umbrella skirt structure along arrester;
3. The umbrella skirt structures are not salient for directional characteristics;
4. The normal of arrester changes regularly.

Therefore, the point cloud registration of lightning arresters is complicated and prone to misregistration. The characteristics of arresters need to be described for the further registration.

#### Conventional registration method

In order to study the registration method of arrester, the representative conventional registration methods include ICP algorithm, LM algorithm, 3D-NDT algorithm, and FPFH descriptorare verified.

**Figure 4:**
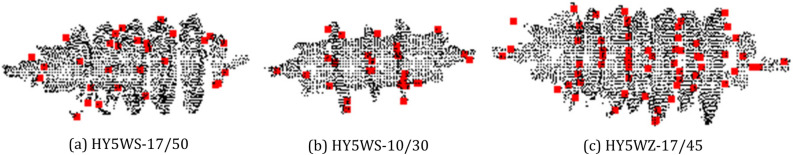
The processing results. The processing results of four conventional point cloud registration methods on typical arrester of Fig. 3.

The registration experiments are conducted on point clouds after these methods are adjusted to the best parameters. The results are shown in Fig. 4.

The shortcomings of these methods are shown below:

1. ICP and LM algorithms are unable to align the umbrella skirt structure.
2. 3D-NDT algorithm has poor registration effects on the smaller arresters.
3. The FPFH descriptor can register the umbrella skirt structure, but it takes too much time.

Therefore, the conventional point cloud registration method can’t meet the requirements of measurement.

### Registration process

We propose a novel SIPFH descriptor, an improved Levenberg-Marquardt (LM) algorithm is also presented. In order to solve the problems of arrester registration, an effective method that includes a primary registration method based on SIPFH and a secondary registration method based on improved LM algorithm are developed.

**Figure 5:**
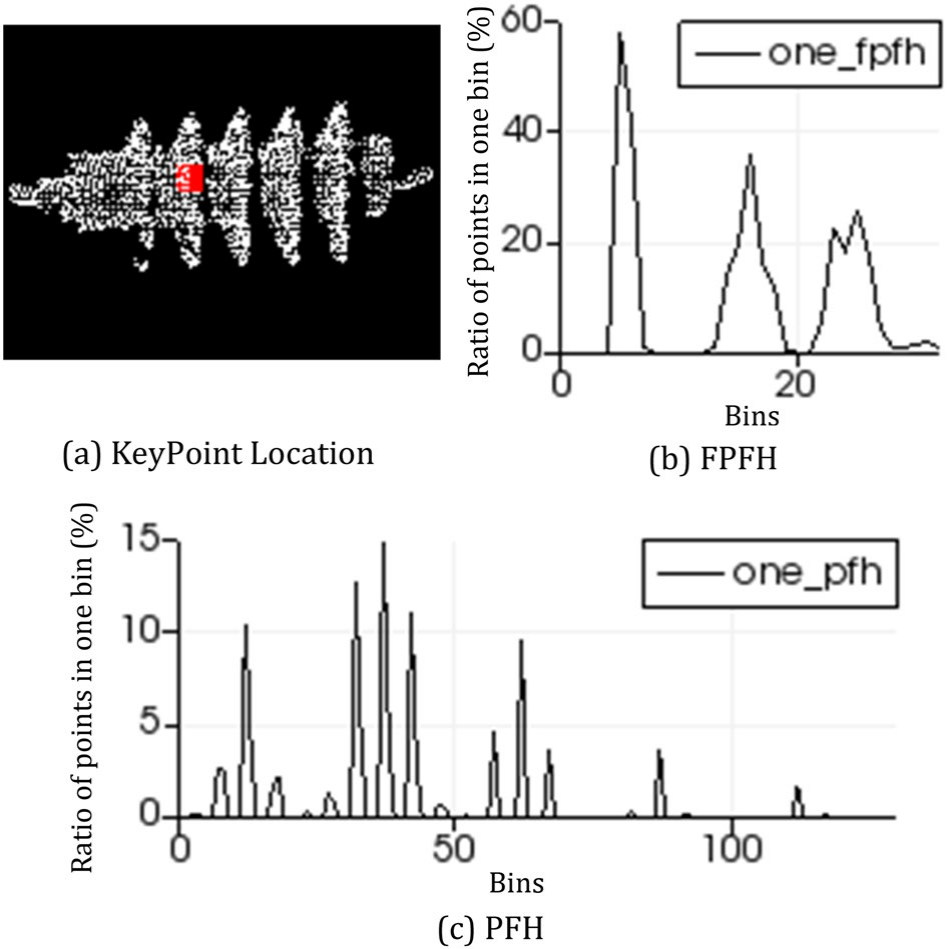
Point cloud registration method based on SIPFH. Firstly, the scale-invariant keypoints are located after generating the point cloud, and then the FPFH features of the keypoints are calculated to obtain the SIPFH descriptor. Secondly, SAC-IA algorithm determines the best matching scheme for two-point cloud and completes the initial point cloud registration. Finally, the improved LM algorithm realize the local optimization of two-point cloud.

The flow of the point cloud registration method for arrester is shown in Fig. 5. The process comprises the following steps:

1. The scale-invariant keypoints of arrester are extracted, and then the FPFH descriptor is calculated at the keypoints to get the scale-invariant point feature histogram (SIPFH). The rigid transformation matrix with the best error metric is determined by SAC-IA algorithm. Finally, the primary registration is completed.
2. The improved LM algorithm realizes the local optimization; the process include the following steps: First, an incremental method equation is constructed to reduce the distance between two point clouds. Then, if the change in this step is smaller than threshold, the maximum space for detecting the corresponding point is reduced; the accuracy of registration is improved during the iteration process. Finally, the secondary rigid transformation matrix is obtained by SVD method.

### Scale-invariant feature transform

Scale-invariant feature transform (SIFT) algorithm is a kind of computer vision algorithm. It contain the following three steps: scale-space extrema detection, orientation assignment, keypoint description^13^. SIFT descriptor has good translation, rotation and scaling invariance, and plays an important role in image mosaic, image retrieval and target recognition^14,15^.

In this paper, 3D-SIFT algorithm is used to extract the feature descriptor of point cloud data. Similar to the performance of 2D image, 3D-SIFT algorithm constructs a local scale-space from the three-dimensional space of the point cloud to detect extreme points. Gaussian pyramid of volume image is built through sequential Gaussian blurring and down-sampling of volume image to make the feature points scale invariant^16^. The definition of scale space by 3D-SIFT is shown in Eq. (1), $G(x,y,z,\sigma )$ is the Gauss kernel function, and *P*(*x*, *y*, *z*) is 3D coordinate of the point cloud.

1 $$\begin{aligned} \begin{aligned} L(x,y,z,\sigma ) = G(x,y,z,\sigma ) * P(x,y,z). \end{aligned} \end{aligned}$$

In order to obtain stable keypoints in the local scale-space, subtracting the two adjacent Gauss kernel function in the same octave to generate Difference-of-Gaussian (DoG)^16^, as shown in Eq. (2). Each voxel is compared with those of its neighbors to find the extremum in DOG. The neighborhoods is only 6 neighbors sharing the face with the interrogated cube, and the two cubes located at the same position in the adjacent DoG scales counts^17^. Some extreme points are unstable and will be filtered.

2 $$\begin{aligned} \begin{aligned} D(x,y,z,k^i \sigma )= P(x,y,z) * (G(x,y,z,k^{i+1} \sigma ) - G(x,y,z,k^i \sigma )). \end{aligned} \end{aligned}$$

These extreme points are approximately equal to the keypoints. Then, the direction of the keypoint is calculated to realize the rotation invariance of 3D objects^17^, the calculation formula is shown in Eq. (3). *m*(*x*, *y*, *z*) is the vector size of the keypoint direction. $\theta (x,y,z)$ is the azimuth of each point to their center $(x_c,y_c,z_c)$. $\phi (x,y,z)$ is the elevation angle.

3 $$\begin{aligned} {\left\{ \begin{array}{ll} m(x,y,z) = \sqrt{(x - x_c)^2 + (y - y_c)^2 + (z - z_c)^2}.\\ \theta (x,y,z) = \tan ^{-1} ((y - y_c)/(x - x_c)).\\ \phi (x,y,z) = \sin ^{-1} ((z - z_c) / m(x,y,z)). \end{array}\right. } \end{aligned}$$

The location and scale of keypoints are accurately determined by fitting two dimensional function. An approximate Harris Corner detector is used to remove the keypoints of low contrast and unstable edge response points^18^. In this way, the stability and the ability to resist noise are enhanced.

**Figure 6:**
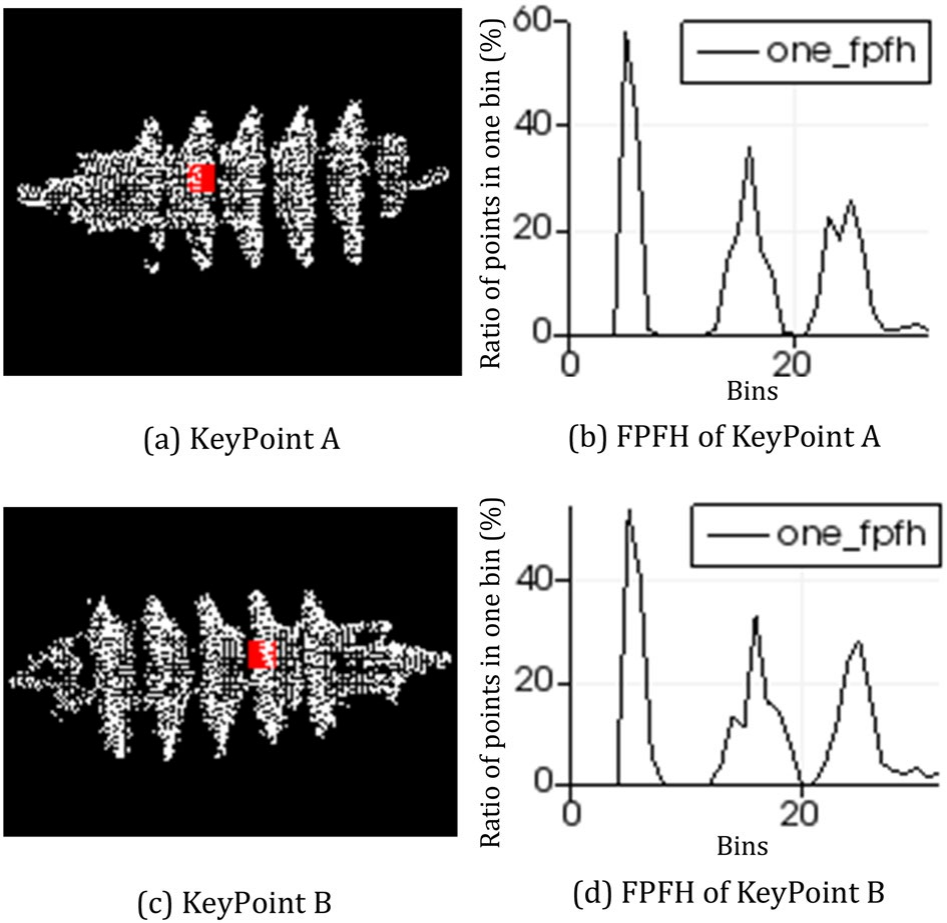
3D-SIFT keypoints of arrester. From top to bottom: 3D scale-invariant keypoints of three typical arresters.

In Fig. 6, 3D scale-invariant keypoints are extracted from point clouds of these three arresters. Its characteristics are as follows:

1. The keypoints are mainly located in the umbrella skirt region and the edge region.
2. The keypoints are evenly distributed on the arrester surface.
3. The keypoints of one object are in the same position.

3D Scale-invariant keypoints have some advantages in the feature description of arrester, which can be used for point cloud registration.

The conventional 3D-SIFT algorithm regard the gradient histograms on the regular icosahedron as descriptors. This descriptor takes much time in the application and requires much higher computational cost.

### Fast point feature histogram

The function of point feature histogram (PFH) is to encode the K-neighborhood geometric characteristics of points by summarizing the average curvature around points^10^. It is invariant to the corresponding surface of point cloud. PFH is robust under different sampling densities or noise in the neighborhood. The PFH calculation of points depends on the existence of 3D coordinates and estimated surface normal. However, the calculation process of PFH is complicated, it’s difficult to achieve real-time point cloud registration.

The process of PFH calculation consists of the following steps. First, the normal *n* of each point is found, and then determine neighborhood radius *R*. Second, all points are regarded as the origin, and select neighbors within the radius to calculate the three elements ($\alpha$, $\phi$, $\theta$) of PFH features,then save these variables and euclidean distance among these points and merge them into a histogram. Finally, the PFH descriptor is a histogram containing the relationship among all point pairs in the neighborhood^10^.

Fast Point Feature Histograms (FPFH) descriptor is simplified from PFH^19^. These steps of the feature description are as follows:

1. Each query point $P_e$ set of tuples $\alpha$, $\phi$, $\theta$ between itself and its neighbors $P_k$ are calculated as described in PFH descriptors; it is called Simplified Point Feature Histogram (SPFH);
2. FPFH is the sum of all SPFH weights.

SPFH calculates three features ($\alpha$, $\phi$, $\theta$) between the query point and its neighbors. There is unnecessary to calculate three elements among all points in the region. The expression for calculating FPFH by weighted nearest neighbor SPFH is:

4 $$\begin{aligned} \begin{aligned} FPFH(P_e) = SPFH(P_e) + \frac{1}{k} \ \sum _{i=1}^k \frac{1}{\omega _k} \ \cdot SPFH(P_k). \end{aligned} \end{aligned}$$

Where *k* is the quantities of points except for the query point in the neighborhood; $P_e$ is the query point; $\omega _k$ is the weight. FPFH depends on the query point and its neighbors on the space.

**Figure 7:**
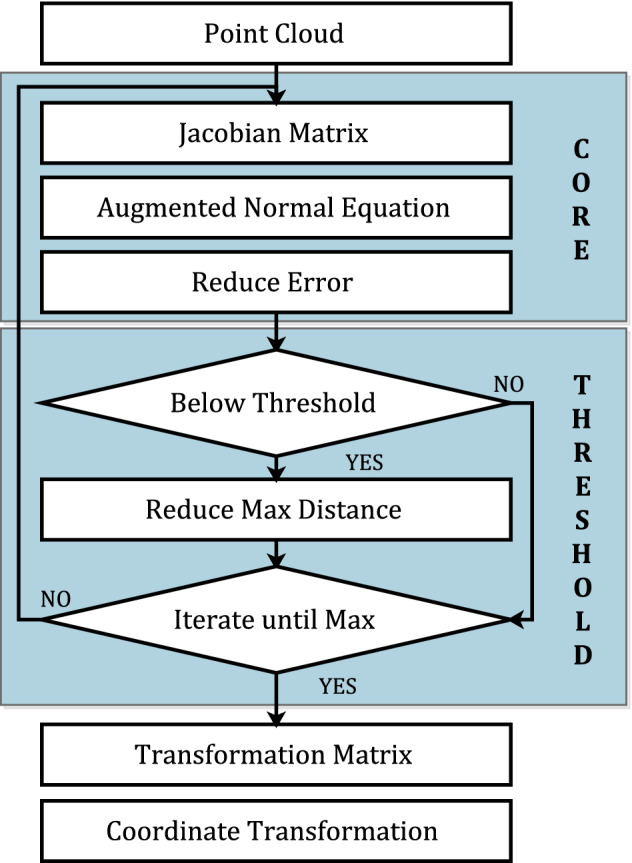
The Feature Descriptors. Figure (**a**) shows a keypoint on the arrester model; The FPFH descriptor of keypoint in figure (**a**) is shown in figure (**b**), and the PFH descriptor is shown in figure (**c**).

The effects of PFH and FPFH at the same keypoint are shown in Fig. 7. FPFH and PFH have the same error metrics in subsequent registration; The running time of FPFH is less than PFH^10^. Therefore, FPFH is used for keypoint description.

The conventional FPFH method is utilized to calculate FPFH descriptor on all points, and thus, it exhausts lots of computational memories and takes longer time for calculation in practical applications.

### SIPFH

The conventional method based on FPFH has some deficiencies. The 3D-SIFT descriptor takes up a lot of computational cost and spends much time in running. Therefore, it seems that FPFH and SIFT keypoints are good choices. In this paper, SIPFH descriptor is proposed to describe the local geometry around keypoint. During the calculation process of descriptor, keypoint extraction and keypoint location are same as that in SIFT^20^. The difference is that SIPFH is a keypoint descriptor. SIPFH combines FPFH and SIFT together.

The calculation process of SIPFH descriptor include the following steps:

1. Calculate scale-invariant keypoints of arrester. As shown in Fig. 6, scale-invariant keypoints are extracted from point clouds of those three types of arresters.
2. Gain FPFH descriptor at keypoints. *d* Independent feature histograms are created, one for each feature dimension, and concatenate them together. As shown in Fig. 7, there are 33-dimensional FPFH and 125-dimensional PFH of the same point for one arrester.
3. Obtain SIPFH descriptors of arresters. Feature dimension of FPFH and the vector size of the key point direction make different contributions to the whole descriptor. So they need to be weighted to describe the keypoint precisely. SIPFH is defined as Eq. (5). Where $d_i$ represents the feature dimension of FPFH, $\omega$ is the weight of vector size of the key point direction. Let $S_i$ denote the feature dimension of SIPFH, where *i* is generally 33. Finally, combine each feature dimension of SIPFH together.

5 $$\begin{aligned} \begin{aligned} S_i = d_i + \omega m. \end{aligned} \end{aligned}$$

Therefore, SIPFH descriptors are extracted with SIFT and FPFH; it has excellent ability of descriptiveness and scale invariance; running time is short. The feature have a computational complexity of *O*(*k*).

### Sample consensus initial algorithm

The sample consensus initial algorithm (SAC-IA) randomly selects some points used to perform random matching; the rotation and displacement are solved by SVD. Then all the remaining matching items are tested to verify whether they meet the polarity constraints obtained from this matrix. SAC-IA attempts to maintain the same geometric relations of the correspondences without the finite correspondence sets^10^. A large numbers of correspondence candidates are sampled and ranked quickly.

The algorithm include the following:

1. The sampling points are selected from point cloud *P* while ensuring sampled points have different FPFH characteristics as much as possible; besides, their distances among sampling points are larger than a user-defined minimum distance *d*;
2. A series of points are selected in target point cloud *Q* for each sample point whose FPFH features are similar to sample points in *P*. In this case, a point is randomly selected as a corresponding point;
3. The rigid transformation matrix is computed by SVD algorithm through sample points and corresponding relationships. Then an error metric is calculated for the point cloud that calculates the transformation quality^10^.

The above three steps are repeated, then all matches of the polarity constraint are computed. Finally, the transformation with the best error metric is selected.

**Figure 8:**
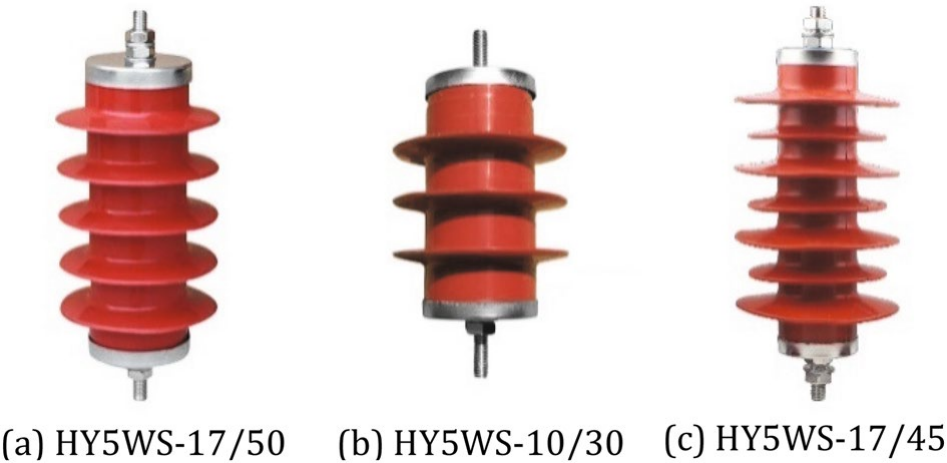
Corresponding point FPFH descriptor. Arrester point cloud in figure (**a**) and figure (**c**) is obtained from different acquisition angles ; Figure (**a**) shows the keypoint A of arrester point cloud; Figure (**b**) shows the FPFH descriptor of keypoint A; Figure (**c**) shows keypoint B of another arrester point cloud; Figure (**d**) shows the FPFH descriptor of keypoint B.

SAC-IA is used to determine the best corresponding point of arrester. FPFH descriptor of the corresponding point is shown as in Fig. 8. The Fig. 8a,c are corresponding points of arrester. Figure 8b,d are 33-dimensional FPFH of the corresponding points. FPFH descriptor of the corresponding points are used as matching point to calculate the rigid transformation matrix in matching algorithm.

SAC-IA relies on SIPFH descriptors to calculate the optimum matching scheme.

### Improved LM algorithm

**Figure 9:**
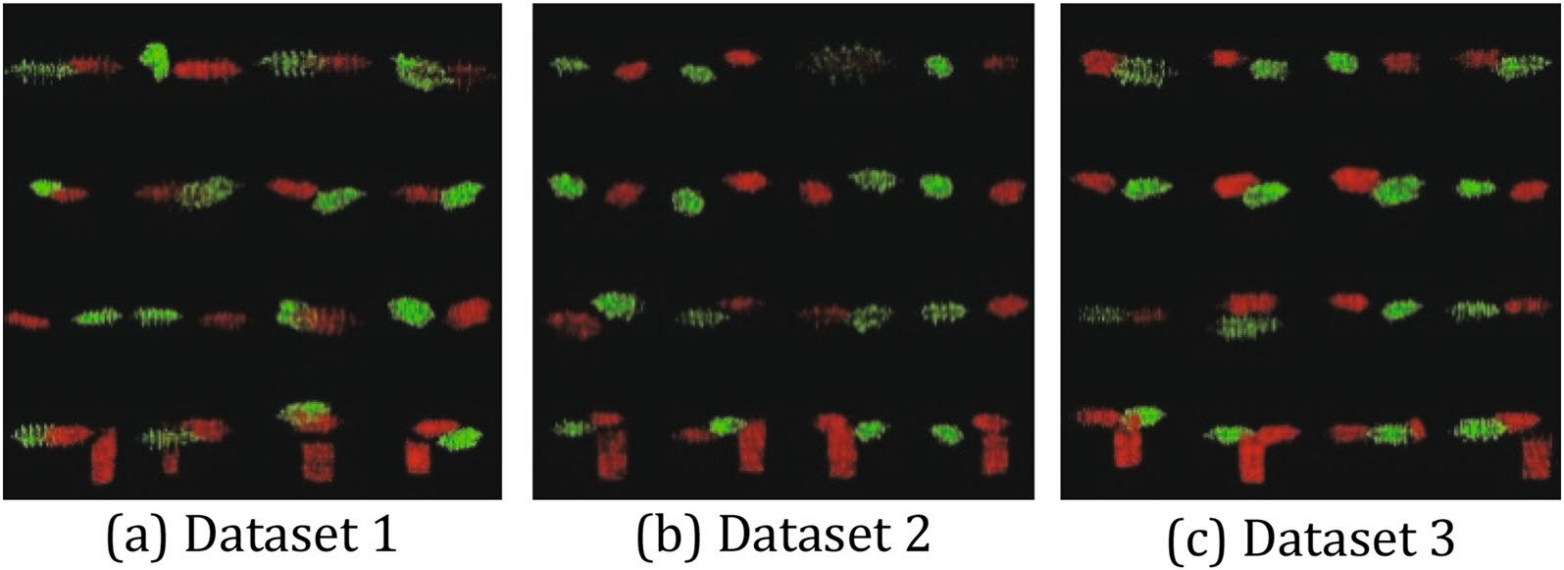
The improved LM algorithm. The improved LM algorithm is made up of two major components: the core component and the threshold component. The core component constructs the augmented normal equation to reduce the error, which is assame as the conventional LM algorithm. The threshold component describes the threshold; If the step change is lower than the threshold, the maximum distance of the corresponding points is reduced to improve the registration accuracy.

The improved LM algorithm can automatically adjust the maximum distance among corresponding points as shown in Fig. 9.

In the core part of Fig. 9, it is consistent with the conventional LM algorithm. The incremental method equation is constructed to reduce the distance between the two point clouds.

LM algorithm is an iterative algorithm to solve non-linear least squares problems. It interpolates between the method of gradient descent and Gauss-Newton (GN) algorithm^9^.

In Eqs. (6)–(8), *P* is an estimator, $P^+$ is the optimal parameter, $\varepsilon$ is the error between observation vector and estimated observation vector, *x* is an observation vector. *f* is function that maps parameter vector *P* to the estimated observation vector $\overset{\frown }{x}$.

6 $$\begin{aligned} P^+ & = \arg \min \varepsilon ^T \varepsilon . \end{aligned}$$

7 $$\begin{aligned} \varepsilon & =  x- \overset{\frown }{x}. \end{aligned}$$

8 $$\begin{aligned} \overset{\frown }{x} & =  f(P). \end{aligned}$$

The Taylor expansion of Eq. (8) is shown in Eq. (9), where *J* is the Jacobian matrix.

9 $$\begin{aligned} f(P+\delta _P) \approx f(P)+ J \delta _P. \end{aligned}$$

At each step, Eq. (10) is minimized by finding step length $\delta _P$. It can be proved that the optimal solution of least-squares exists when $J \delta _P-\varepsilon$ is orthogonal to *J*. This leads to $J^T(J \delta _P -\varepsilon )=0$, and Eq. (11) is obtained, which yields $\delta _P$ the solution of the so-called normal equations^21^.

10 $$\begin{aligned}{}&\begin{aligned} \begin{Vmatrix} x-f(P+\delta _P) \end{Vmatrix}&\approx \begin{Vmatrix} x-f(P)-J \delta _P \end{Vmatrix} = \begin{Vmatrix} \varepsilon -J \delta _P \end{Vmatrix}. \end{aligned} \end{aligned}$$

11 $$\begin{aligned}&J^T J \delta _P = J^T \varepsilon . \end{aligned}$$

The normal incremental equation of the LM algorithm introduces a damping term $\mu$ where *I* is an identity matrix, as shown in Eq. (12).

12 $$\begin{aligned} (J^T J + \mu I) \delta _P = J^T \varepsilon . \end{aligned}$$

If the currently evaluated $\delta _P$ reduces the error, this update is accepted, then the process is repeated to reduce the damping term $\mu$. Otherwise, the damping term is increased, the augmented normal equations are solved again, and the process is iterated until the value $\delta _P$ that decreases error is found^22^. In LM, each iteration of this algorithm will adjust the damping term to reduce the error^21^.

The conventional LM algorithm falls into the optimal local solution quickly when the point cloud position is close. At the same time, it may obtain wrong results, and will reduce the accuracy of the transformation matrix.

In this paper, LM algorithm is used to solve the problems of arrester registration when the position and attitude are close between two point clouds. In addition, the algorithm does not reduce the registration accuracy. Therefore, a threshold $\omega$ is introduced in the improved LM algorithm. As the process iterates, if the step changes $J^T \varepsilon$ are less than the threshold, the maximum distance between corresponding points is reduced to improve the accuracy of registration. Then, the transformation matrix will be adjusted, as shown in the threshold part of Fig. 9. When the direct distance between two point clouds is unknown, the registration accuracy of the algorithm is robust.

After experiments, the improved LM algorithm can improve the registration accuracy as high as possible without occupies lots of memory. In extreme cases, it does not reduce the registration accuracy.

## Results

In this section, experiments are based on the Intel(R) Core (TM) i5-5200U CPU @ 2.20GHz hardware platform and Microsoft Visual Studio 2019 software platform. The algorithms are all applied by the C++ programming language, and the PCL-1.11.0 point cloud library is used.

### Evaluation index

Three essential indicators are introduced to evaluate the accuracy of the rigid transformation matrix, including average error (AME), root mean square error (RMSE), and standard deviation (SD)^23^. As shown in Eqs. (13)–(15).

13 $$\begin{aligned} E_{arg} & =  \frac{1}{N} \begin{vmatrix} \sum (R_i-R_j) \end{vmatrix}. \end{aligned}$$

14 $$\begin{aligned} RMSE&=  \sqrt{\frac{1}{N} \sum _{i=1}^N {(x_i)^2}}. \end{aligned}$$

15 $$\begin{aligned} \sigma & = \sqrt{\frac{1}{N} \sum _{i=1}^N {(x_i-\mu )^2}}. \end{aligned}$$

In the equations: $R_i$ is the standard transformation matrix. $R_j$ is the transformation matrix obtained from the registration algorithm. *N* is the total number of elements from the transformation matrix. $x_i$ is the absolute value of the difference between elements, obtained from $R_i$ and $R_j$. $\mu$ is the mean of $x_i$. The standard transformation matrix is calculated by the transformation between rotation and translation of laser camera^24^, the calculation formula is shown in Eq. (16). $\alpha$ is the angle in which the coordinate system *B* rotates counter-clockwise about the x-axis of the coordinate system *A*. The amount of translation of coordinate system *B* along each axis of coordinate system *A* is:$T_{x_x}$, $T_{x_y}$, $T_{x_z}$.

16 $$\begin{aligned} P_a = { \begin{bmatrix} 1 &{} 0 &{} 0 \\ 0 &{} \cos (\alpha ) &{} -\sin (\alpha ) \\ 0 &{} \sin (\alpha ) &{} \cos (\alpha ) \end{bmatrix} } P_b + { \begin{bmatrix} T_{x_x}\\ T_{x_y}\\ T_{x_z} \end{bmatrix} } = R_x P_b + T_x. \end{aligned}$$

### Accuracy evaluation experiment

In this section, the experiments select the registration methods proposed by paper^5^, paper^9^, paper^10^, and paper^16^ to compare with ours.

- ICP-v2: ICP algorithm using point-to-point and point-to-surface measurement functions as a probability framework;
- LM: The conventional LM algorithm;
- FPFH: The point cloud registration method based on FPFH descriptor and SAC-IA algorithm.
- 3D-SIFT: The gradient histograms on the regular icosahedron is calculated.

**Figure 10:**
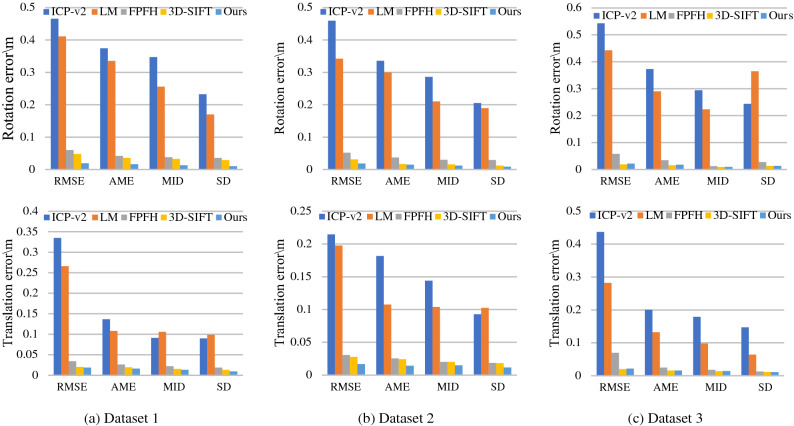
Registration error comparison. Figure (**a**) from top to bottom: rotation rigid transformation error and translation rigid transformation error in dataset 1. Similarly, figure (**b**) is the rigid transformation error in dataset 2; Figure (**c**) is the rigid transformation error in dataset 3.

We conducted experiments on registration error of point cloud on three datasets. The evaluating indicators include: RMSE, AME, median error (MID), SD, minimum error (MIN), and maximum error (MAX). In Fig. 10, the figure (a) from top to bottom are rotation rigid transformation error and translation rigid transformation error in dataset 1; Similarly, figure (b) is the rigid transformation error in dataset 2; Figure (c) is the rigid transformation error in dataset 3. As shown in Fig. 10, the registration errors of our method are lower than other methods; our method are less than 0.025 m in RMSE. In Table 1, the total error of lightning arrester point cloud registration in three datasets; the RMSE of ours is 0.019 m.

**Table 1:** The total error of rigid transformation for point cloud registration in three datasets (unit: m).

ALGO	RMSE	AME	MID	SD	MIN	MAX
ICP-v2	0.409	0.222	0.473	0.169	0.031	2.237
LM	0.324	0.166	0.321	0.165	0.026	2.031
FPFH	0.051	0.029	0.050	0.024	0.010	0.790
3D-SIFT	0.028	0.021	0.018	0.016	0.007	0.434
Ours	0.019	0.016	0.026	0.011	0.006	0.385

**Figure 11:**
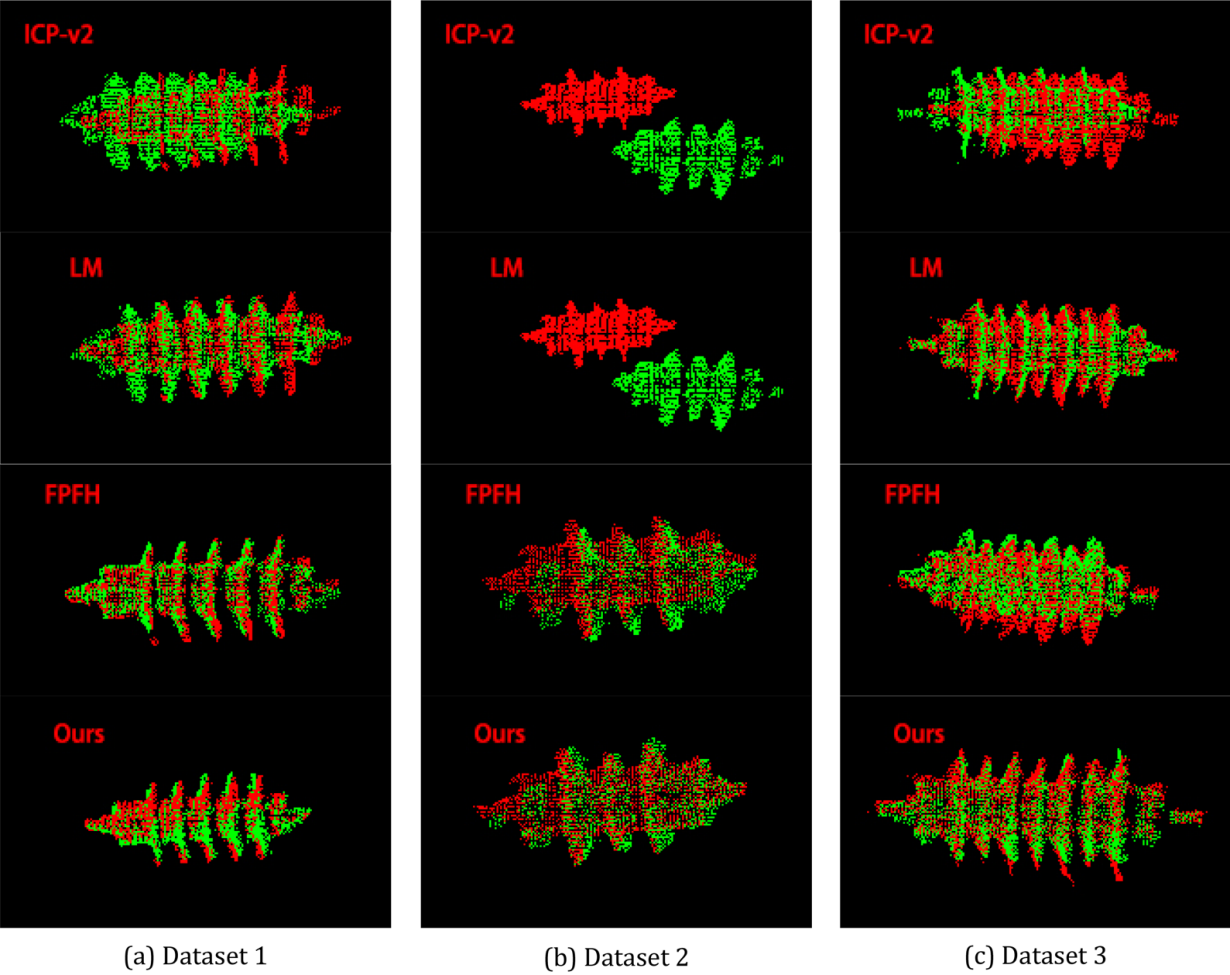
Arrester registration results. Figure (**a**) from top to bottom: registration results of icp-v2, LM, FPFH, 3D-SIFT and ours in dataset 1. Similarly, figure (**b**) is the registration result of dataset 2; Figure (**c**) is the registration result of dataset 3.

In Fig. 11, Registration results of these algorithms. In ICP-v2 and LM, the registration errors are relatively large. In Fig. 11b, ICP-v2 and LM methods fail, since it is impossible to register arresters with inconspicuous characteristics. The FPFH method can register the umbrella skirt structure by FPFH descriptor. However, its registration accuracy is lower than ours. The registration errors of 3D-SIFT method is well.

### Computing time

**Figure 12:**
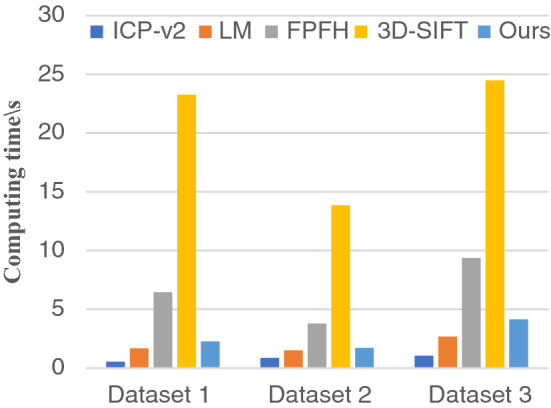
Average computing time. The average computing time of point cloud registration in three datasets.

In this experiment, the average running time is counted for four registration methods, as shown in Fig. 12. The average running time of this paper is 2.7 s, which is $41.4\%$ of the FPFH method.

The time of our method is much shorter than the conventional registration method based on FPFH; our method can achieve real-time point cloud registration results with low CPU performance.

## Discussion

The experimental results in “Materials and methods” section showed that our method is better than the conventional point cloud registration methods.

ICP-v2 algorithm^5^ and LM algorithm^9^ are weak in describing point cloud features. These algorithms are easy to fall into locally optimal solutions, which is reflected in the dislocation of umbrella skirt structure of the arrester. The RMSE error of ICP algorithm is 0.409 m; the RMSE error of LM algorithm is 0.324 m.

The method based on FPFH^10^ is effective; the RMSE error of FPFH algorithm is 0.051 m. However, the calculation speed is slow; the registration efficiency is low; the average operation time is 6.52 s. This method can reduce the running time only on the 8-core system through multithreading, which is inconvenient.

The method based on 3D-SIFT^16^ is well; but the average operation time is 20.54 s. Our method is much shorter than FPFH and 3D-SIFT method, and thus, can achieve real-time point cloud registration on systems with low CPU cost.

The secondary registration alglrithm based on improved LM is significant to improve the registration accuracy. The transformation matrix obtained by SAC-IA is prone to errors. Therefore, the improved LM algorithm can realize local optimization as much as possible.

**Figure 13:**
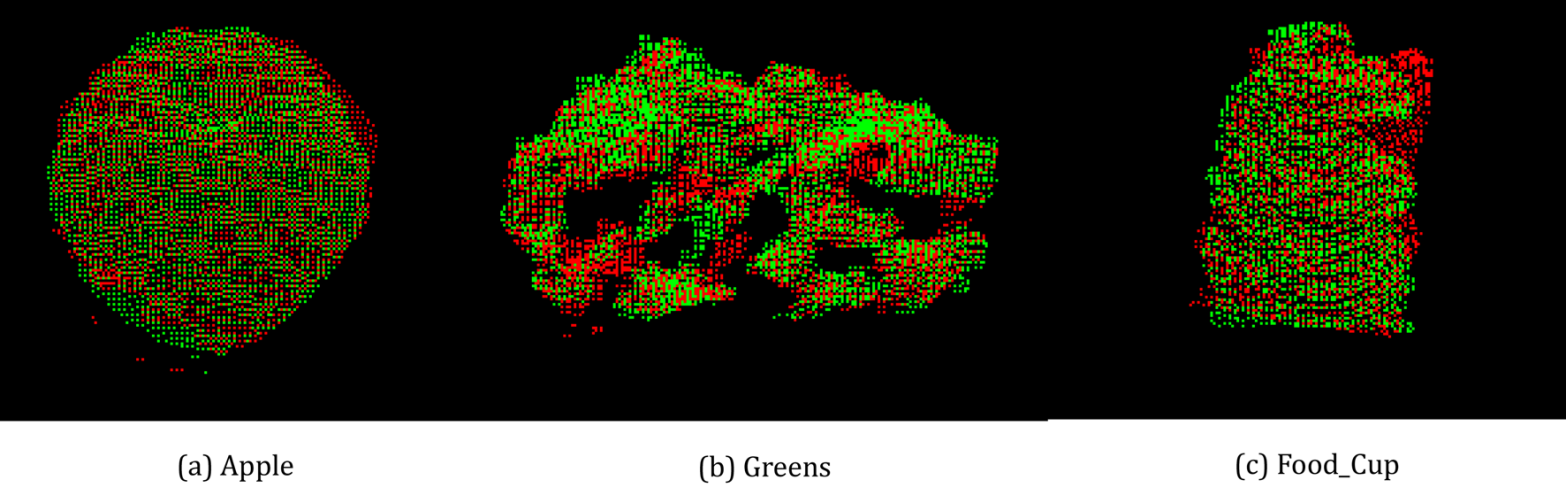
The other registration results. The registration results of some point clouds on “RGB-D Object Dataset”.

In addition, we test our method on “RGB-D Object Dataset”^25^, and some results are shown in Fig. 13. The better performance of ours is achieved in terms of the registration results.

The main parameters of arrester include: creepage distance, umbrella distance, and the diameter. When whole 3D information of the arrester is obtained, the umbrella distance can be obtained by calculating the distance among the nearest points in the edge of the umbrella skirt structure; the creepage distance can be obtained by calculating the shortest distance along the surface among the interface parts.

## Conclusions

It is effective to detect the structural parameters of arrester by 3D reconstruction. In this paper, a SIPFH descriptor is proposed to solve the description problem of umbrella skirt structure, and to improve the running efficiency. We also present an improved LM algorithm for local optimization of point cloud registration. Furthermore, we established an effective method structure that includes a primary registration method based on SIPFH and a secondary registration method based on an improved LM algorithm.

Experimental results show the high registration accuracy. The RMSE of ours is 0.019 m. The average running time is 2.7 s, which is $41.4\%$ of the conventional FPFH registration method. It can realize real-time registration on systems with lower CPU.

## Data Availability

Data underlying the results presented in this paper are not publicly available at this time but may be obtained from the corresponding author upon reasonable request.
